# Longitudinal change in c-terminal fibroblast growth factor 23 and outcomes in patients with advanced chronic kidney disease

**DOI:** 10.1186/s12882-021-02528-2

**Published:** 2021-10-02

**Authors:** Helen V. Alderson, Rajkumar Chinnadurai, Sara T. Ibrahim, Ozgur Asar, James P. Ritchie, Rachel Middleton, Anders Larsson, Peter J. Diggle, Tobias E. Larsson, Philip A. Kalra

**Affiliations:** 1 Vascular Research Group, Manchester Academic Health Sciences Centre, University of Manchester, Salford Royal NHS Foundation Trust, Salford, UK; 2 Department of Renal Medicine, Salford Royal Hospital, Level 2, Hope Building, Stott Lane, Salford, M6 8HD UK; 3 Department of Internal Medicine and Nephrology, Faculty of Medicine, Alexandria University, Alexandria, Egypt; 4 Department of Biostatistics and Medical Informatics, Acibadem Mehmet Ali Aydinlar University, Istanbul, Turkey; 5 Section of Clinical Chemistry, Department of Medical Sciences, Uppsala University, Uppsala, Sweden; 6 CHICAS, Lancaster Medical School, Lancaster University, Lancaster, UK; 7 Department of Clinical Science, Intervention and Technology, Renal Unit, Karolinska Institute, Stockholm, Sweden

**Keywords:** cFGF23, CKD, Survival outcomes

## Abstract

**Background:**

Fibroblast growth factor23 (FGF23) is elevated in CKD and has been associated with outcomes such as death, cardiovascular (CV) events and progression to Renal Replacement therapy (RRT). The majority of studies have been unable to account for change in FGF23 over time and those which have demonstrate conflicting results. We performed a survival analysis looking at change in c-terminal FGF23 (cFGF23) over time to assess the relative contribution of cFGF23 to these outcomes.

**Methods:**

We measured cFGF23 on plasma samples from 388 patients with CKD 3-5 who had serial measurements of cFGF23, with a mean of 4.2 samples per individual. We used linear regression analysis to assess the annual rate of change in cFGF23 and assessed the relationship between time-varying cFGF23 and the outcomes in a cox-regression analysis.

**Results:**

Across our population, median baseline eGFR was 32.3mls/min/1.73m^2^, median baseline cFGF23 was 162 relative units/ml (RU/ml) (IQR 101-244 RU/mL). Over 70 months (IQR 53-97) median follow-up, 76 (19.6%) patients progressed to RRT, 86 (22.2%) died, and 52 (13.4%) suffered a major non-fatal CV event. On multivariate analysis, longitudinal change in cFGF23 was significantly associated with risk for death and progression to RRT but not non-fatal cardiovascular events.

**Conclusion:**

In our study, increasing cFGF23 was significantly associated with risk for death and RRT.

**Supplementary Information:**

The online version contains supplementary material available at 10.1186/s12882-021-02528-2.

## Background

Fibroblast growth factor-23 (FGF23) is a regulatory hormone produced by osteocytes and osteoblasts; it plays a significant role in phosphate homeostasis [1]. FGF23 is up regulated early in CKD and functions as an important regulator of phosphate, promoting phosphaturia and reducing the production of 1,25-dihydroxyvitamin D [2]. Recognition of FGF23 as an important pathological factor in CKD has made it a prime biomarker to investigate in clinical studies and a potential target for future therapeutic intervention [3, 4].

Numerous studies have investigated the relationship between baseline FGF23 and clinical outcomes in CKD. FGF23 has been shown to associate with risk for death [5–10] and cardiovascular events [7, 9, 11–13] in both dialysis and non-dialysis populations.

There is growing understanding of the underlying pathophysiological mechanisms. FGF23 causes reversible hypertrophy of cardiac myocytes in mice [14] and in myocardial autopsy samples from dialysis patients [15]. High levels of FGF23 are positively correlated with increasing blood pressure [16] and pulse pressure [17] in CKD, this may be due to impairment of endothelial dependent vasorelaxation [18], High levels of FGF23 are associated with arterial stiffness [19], atherosclerosis [19, 20] and aortic valve calcifications [21] in CKD. FGF23 can directly stimulate the hepatic production of inflammatory cytokines such as C reactive protein [22]. High FGF23 impairs host defense in mice and CKD patients [23], increasing risk of infection related hospitalizations in older adults [24].

Elevated levels of FGF23 have been associated with risk for progression of kidney disease and renal replacement therapy [7–10, 25, 26] and in non-CKD populations they have been shown to predict incident CKD [27, 28]. More recent studies have investigated whether FGF23 can improve reclassification of risk [29].

Although these clinical studies have identified that FGF23 is an important risk factor in CKD, the majority do not account for change in FGF23 over time. There is individual variability in FGF23 levels but studies in both healthy participants [30] and CKD patients have shown that high levels confer increased risk [31].

Three studies have sought to investigate change in FGF23 over time and the effect on risk for death in CKD patients [5, 7, 8]. The study by de Bouma et al. showed that the rate of change in FGF23 was not predictive of mortality, whereas the study by Isakova et al. demonstrated that rapidly rising FGF23 levels conferred an increased risk for death in comparison to stable levels.

Our study examined cFGF23 levels over time in a cohort of 388 patients selected from the Salford Kidney Study (SKS) to analyse the relationship between change in cFGF23 and the clinical outcomes of death, renal progression and cardiovascular events.

## Methods

### Patient population

The Salford kidney study (SKS), Salford, UK is a prospective observational study to investigate outcomes in kidney disease. Three hundred eighty-eight subjects recruited between 2004 and 2013 who had sufficient stored baseline and serial plasma samples were selected for this analysis. Details of the SKS cohort (previously known as the chronic renal insufficiency standards implementation study, CRISIS) have been published previously [32–35]. In brief, patients aged 18 years or older who are referred to our renal center for management of CKD and have an eGFR≤60mls/min/1.73m^2^ are eligible for recruitment. Patients are managed in accordance with standard clinical practice guidelines and are followed up until death or initiation of renal replacement therapy (dialysis or transplantation). Demographic data are recorded at baseline and annually during study follow up. Blood samples are drawn for standard clinical tests and additional samples stored for subsequent biomarker analyses. Three hundred eighty-eight patients had repeated measurements of cFGF23, with a mean of 4.2 measurements per individual. Time zero was defined as the baseline sample date and follow-up was until death, initiation of renal replacement therapy, or April 2014. Date and cause of death was obtained from the Office of National Statistics (ONS), date of initiation of renal replacement therapy (RRT) was obtained from the electronic patient records. Major adverse cardiovascular events were recorded during study follow-up and defined as myocardial infarction, coronary angiogram plus angioplasty or stenting, coronary artery bypass graft surgery or stroke. Any patient reported events were verified against primary care and hospital records to ensure accuracy of reporting.

### Biomarker analysis

All biomarker analyses were performed on lithium-heparin plasma samples that had been stored at − 80 °C. cFGF23 was measured in 2014 using a 2nd generation two-site enzyme linked immunosorbent assay (ELISA) supplied by Immutopics (San Clemente, CA, USA) detecting intact FGF23 and its c-terminal fragments. eGFR was calculated from IDMS calibrated creatinine measurements using the 4-variable Modified Diet in Renal Disease (MDRD) study equation [36].

### Statistical analysis

The annual rate of change of cFGF23 (delta cFGF23) and the annual rate of change in estimated glomerular filtration rate (delta eGFR) were calculated by linear regression analysis using all available values between study baseline and endpoint. The cohort was split into four quartiles based on delta cFGF23 values The baseline characteristics and outcomes were compared across these cFGF23 quartiles. Continuous variables were expressed as median (IQR), and *p*-value of the difference between the groups was estimated by Kruskal Wallis H test. Categorical variables were expressed as numbers (%) and p-value of the difference between the groups being calculated by the Chi-square test.

Univariate and multivariate Cox-regression analysis was used to evaluate the strength of association between the cFGF23 quartiles and the outcomes RRT, all-cause mortality prior RRT, and non-fatal cardiovascular events. The univariate analysis included all baseline characteristics, while multivariate models incorporated factors that were significant in the univariate models in a stepwise approach.. Kaplan Meier curves were used to demonstrate the difference in survival patterns between the quartiles with the log-rank test used to test the statistical significance. Throughout the analysis, a *p*-value < 0.05 was taken to be statistically significant. All analysis was performed using SPSS- Version 24 registered to the University of Manchester.

## Results

### Summary data

Baseline demographics for the cohort stratified according to quartiles of delta cFGF23 are presented in Table 1. The median age was 62 years (IQR 52-71), 63.8% of the patients were male, 96% were Caucasian and 25% were diabetic, 52 (13.4%) patients had suffered a prior major cardiovascular event. The median baseline MDRD eGFR was 32.3 ml/min/1.73m^2^. The median study follow up was 70 months (IQR 53 to 97 months).

**Table 1:** Baseline demographic data and outcomes stratified by quartiles of delta FGF23 value

Variables			Delta cFGF23 (RU/mL/year)			
	Total 388	Lower quartile (Q1) (97)	Lower middle quartile (Q2) (97)	Upper middle quartile (Q3) (97)	Upper quartile (Q4) (97)	*p*-Value
Delta cFGF23 RU/mL/year	11.9 (−3 to 57.6)	−13.9 (− 32.3 to −6.7)	3.3 (0.75 to 6.7)	26.4 (17.5 to 37.1)	168.5 (85.4 to 407.2)	**< 0.001**
Age, years	62 (52-71)	62 (50-71)	62 (52-71)	64 (55-70)	61 (52-71)	0.968
Sex, (Male)	245 (63.1%)	56 (57.7%)	70 (72.2%)	58 (59.8%)	61 (63%)	0.166
Ethnicity, (Caucasian)	370 (95.4%)	93 (95.9%)	91 (93.8%)	94 (96.9%)	92 (94.8%)	0.623
Current or former smoker	226 (58.2%)	52 (53.6%)	51 (52.6%)	58 (59.8%)	65 (67%)	0.151
Diabetes	97 (25%)	21 (21.6%)	22 (22.7%)	27 (27.8%)	27 (27.8%)	0.639
Prior cardiovascular event	84 (21.6%)	21 (21.6%)	22 (23.7%)	20 (20.6%)	21 (21.6%)	0.963
Heart failure at baseline	36 (9.3%)	11 (11.3%)	9 (9.3%)	9 (9.3%)	7 (7.2%)	0.806
Mean baseline systolic blood pressure (mmHg)	135 (123-150)	133 (117-150)	135 (123-150)	139 (129-152)	133 (123-150)	0.183
Primary renal disease						
Diabetic nephropathy	52 (13.4%)	12 (12.4%)	5 (5.2%)	16 (16.5%)	19 (19.6%)	
Hypertension	48 (12.4%)	11 (11.3%)	12 (12.4%)	15 (15.5%)	10 (10.3%)	
Renovascular disease	49 (12.6%)	16 (16.5%)	15 (15.5%)	7 (7.2%)	11 (11.3%)	
Glomerulonephritis	87 (22.4%)	29 (29.9%)	23 (23.7%)	16 (16.5%)	19 (19.6%)	**0.004**
ADPKD	32 (8.2%)	5 (5.2%)	4 (4.1%)	11 (11.3%)	12 (12.4%)	
Other	59 (15.2%)	10 (10.3%)	24 (24.7%)	14 (14.4%)	11 (11.3%)	
Unknown	28 (7.21%)	6 (6.2%)	9 (9.3%)	7 (7.2%)	6 (6.2%)	
Baseline laboratory results						
cFGF23 (RU/mL)	162 (101-244)	218 (129-332)	103 (78-160)	141 (100-182)	211 (157-321)	**< 0.001**
Creatinine (mmol/L)	180 (140-241)	189 (140-252)	157 (126-188)	169 (133-213)	227 (181-290)	**< 0.001**
MDRD eGFR (mL/min/1.73m^2^)	32.3 (22.9-43.3)	30 (21-45)	41.2 (30.8-51.7)	33.4 (26.1-41.8)	24.5 (18.8-33.7)	**< 0.001**
Phosphate (mmol/L)	1.09 (0.95 − 1.25)	1.07 (.094-1.27)	1.02 (0.88-1.16)	1.09 (0.93-1.24)	1.16 (1.01-1.31)	**0.001**
Calcium (mmol/L)	2.25 (2.17-2.32)	2.27 (2.18-2.34)	2.25 (2.18-2.31)	2.25 (2.16-2.32)	2.23 (2.14-2.31)	0.198
Albumin (g/L)	44 (42-46)	45 (42-46)	44 (42-46)	44 (41-46)	43 (41-44)	**< 0.001**
Haemoglobin (g/L)	128 (117-140)	126 (116-137)	134 (124-143)	130 (120-141)	122 (111-132)	**< 0.001**
PTH (pg/mL)	54 (31-101)	54 (30-94)	35.5 (25-59.5)	50 (33-101)	90 (52-178)	**< 0.001**
CRP (mg/L)	3.35 (1.4-6.87)	2.8 (1.4-5.95)	2.9 (1.5-5.5)	3.9 (1.4-8.7)	3.6 (1.4-8.8)	0.333
Urinary protein (g/24 h)	0.15 (0.06-0.43)	0.11 (0.06-0.33)	0.12 (0.06-0.30)	0.12 (0.05-0.44)	0.31 (0.16-0.57)	**< 0.001**
Outcomes						
Delta eGFR mL/min/1.73m^2^/year	-1.2 (−2.76 to 0.02)	−0.08 (−0.09 to 0.97)	−0.62 (−1.87 to 0.27)	−1.6 (−3.1 to −0.54)	−2.68 (−4.6 to −1.5)	**< 0.001**
Death prior to RRT (%)	86 (22.2%)	15 (15.5%)	13 (13.4%)	26 (26.8%)	32 (33%)	**0.002**
RRT (%)	76 (19.6%)	8 (8.2%)	6 (6.2%)	13 (13.4%)	49 (50.5%)	**< 0.001**
Total CV events during Follow-up (%)	52 (13.4%)	10 (10.3%)	9 (9.3%)	19 (19.6%)	14 (14.4%)	0.138
Follow up time (months)	70 (52.6-96.8)	69 (53-97)	89 (59-101)	83 (55-101)	57 (43-75)	**< 0.001**

Continuous variables are expressed as median (IQR) and *p* value of the difference between the groups by Kruskal Wallis H test. Categorical variables are expressed as numbers (%) and *p* value of the difference between the groups by Chi- square test

*ADPKD* autosomal dominant polycystic kidney disease, *PTH* parathormone, CRP-c-reactive protein, *cFGF23* Fibroblast growth factor23, Cardiovascular (CV) event- defined as non-fatal stroke or myocardial infarction, coronary angiogram plus angioplasty or stenting, coronary artery bypass graft surgery. Heart failure defined as left ventricular ejection fraction ≤50% or diastolic dysfunction on echocardiogram or a clinical diagnosis of heart failure with no other alternative cause for symptoms. RRT- renal replacement therapy, eGFR-estimated glomerular filtration rate by Modification of Diet in Renal Disease (MDRD) equation

### cFGF23 measurement

Three hundred eighty-eight patients had 3 or more serial measurements of cFGF23. The mean number of measurements per individual was 4.2 (SD 1.5, range 3-9) with a median time of 1.1 years between successive measurements. Across the whole cohort the median baseline level of cFGF23 was 162 RU/ml (IQR 101-244 RU/mL).

### Survival analysis

There was a high rate of progression to RRT in our study, with 76 (19.6%) of patients reaching this endpoint. There were 86 (22.2%) deaths prior to initiation of RRT. During follow-up, 13.4% of the cohort suffered at least one non-fatal major cardiovascular event.

### Rate of change in FGF23 and outcomes

**Fig. 1:**
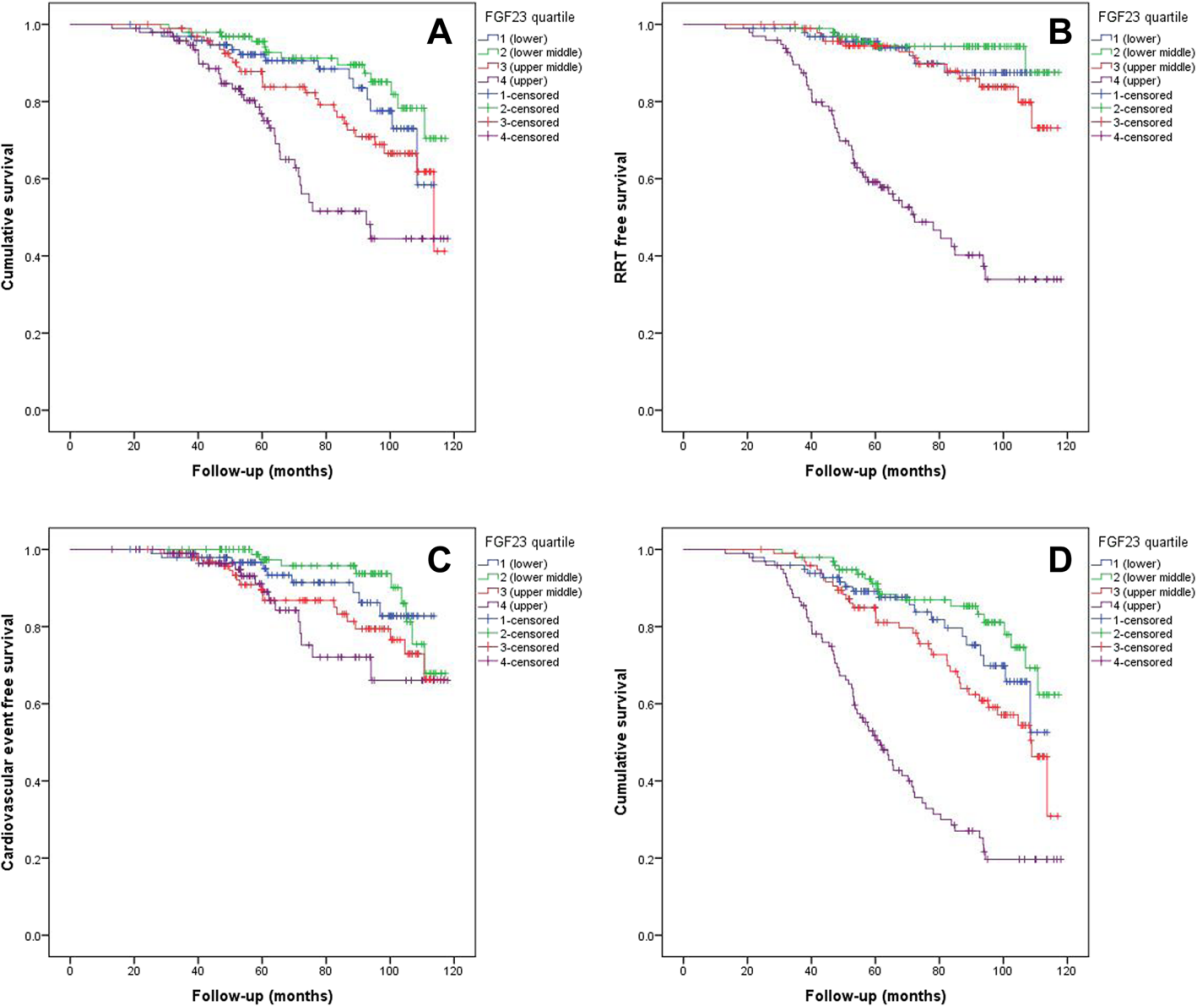
Kaplan Meier graphs for event across quartiles of cFGF23. **A** All-cause mortality prior to renal replacement therapy. **B** Renal replacement therapy free survival. **C** Cardiovascular event free survival. **D** Combined outcome (renal replacement therapy and mortality)

When quartiles of delta cFGF23 were compared to clinical outcomes, there was a significant difference in risk for death, RRT and delta eGFR between those in the highest and lowest quartiles (*p* = 0.002, *p* < 0.001 and *p* < 0.001 respectively) (Table 1). There was no significant difference in non-fatal major cardiovascular events between quartiles of delta cFGF23. There were insufficient data available on cause of death to look at associations with fatal cardiovascular events. In fully adjusted multivariate Cox-regression analysis there was a significant association between higher delta cFGF23 and risk for death (HR 1.46, 95% CI 1.16-1.83, *p* = 0.001) and renal replacement therapy (HR 2.06, 95% CI 1.60-2.67, *p* < 0.001), although there remained a strong inverse correlation between change in eGFR and cFGF23. There was no significant association between delta cFGF23 and non-fatal cardiovascular events in our analysis (HR 1.31, 95% CI 0.99-1.74, *p* = 0.053). Results of the Cox regression analyses in respect to these outcomes are shown in Table 2 and Supplementary Tables 1, 2 and 3. Kaplan-Meier curves in respect to each outcome are shown in Fig. 1.

**Table 2:** Cox-regression models for outcomes

	All-cause mortality prior RRT		Renal Replacement Therapy (RRT)		Cardiovascular event	
	HR (95% CI)	*p*-Value	HR (95% CI)	*p*-Value	HR (95% CI)	*p*-Value
**Univariate model**						
Quartile 1	Reference	–	Reference	–	Reference	–
Quartile 2	0.65 (0.31-1.38)	0.267	0.63 (0.22 -1.82)	0.40	0.72 (0.28-1.84)	0.493
Quartile 3	1.13 (0.87-1.65)	0.270	1.15 (0.74-1.79)	0.54	1.29 (0.86-1.94)	0.207
Quartile 4	1.43 (1.16-1.75)	0.001	2.01 (1.56-2.56)	< 0.001	1.31 (0.99-1.74)	0.053
**Multivariate model 1**						
Quartile 1	Reference	–	Reference	–		
Quartile 4	1.46 (1.16-1.83)	0.001	2.06 (1.60-2.6)^a^	< 0.001		
**Multivariate model 2**						
Quartile 1	Reference	–	Reference	–		
Quartile 4	1.49 (1.18-1.89)	0.001	2.34 (1.57-3.47)	< 0.001		

*HR* hazard ratio, *CI* confidence interval

Multivariate model 1: adjusted for clinical variables at baseline (age, smoker, diabetes, cardiovascular events, heart failure, systolic blood pressure), and cFGF23 quartiles

Multivariable model 2: adjusted for laboratory variables at baseline (MDRD eGFR, phosphate, albumin, haemoglobin, parathormone, cFGF23) and cFGF23 quartiles

^a^Multivariate model 1: adjusted for clinical variables (age, gender), and cFGF23 quartiles

## Discussion

We investigated the relationship between change in cFGF23 over time and risk for death, progression to RRT and cardiovascular events. In our study cFGF23 was associated with risk for death, declining eGFR and RRT, but not with non-fatal cardiovascular events.

Numerous studies have shown baseline cFGF23 to associate with risk for RRT [9, 10]. We have previously demonstrated this in our own study population [26]. cFGF23 is highly correlated with creatinine, after adjustment for this parameter there remained a significant association between delta cFGF23 and risk for RRT. It is noteworthy that our population had advanced CKD and there was a high rate of progression to RRT. In studies that have considered the ability of baseline cFGF23 to predict progression to RRT over a fixed time frame, this marker has failed to improve model fit or discrimination with regards to this outcome measure [29].

cFGF23 was independently associated with risk for all-cause mortality in our study. These findings are consistent with analyses examining the association between baseline FGF23 and risk for death [8–10]. In studies that have looked specifically at improvements in model discrimination with the addition of FGF23, there has been an improvement in outcome prediction and reclassification with the addition of this biomarker, suggesting that it may be a valuable tool for identifying those at greatest risk [29]. The three published studies to have measured serial FGF23 have found that an increasing FGF23 is an independent risk factor for mortality [5, 7, 8] and our study supports these findings.

Other studies have shown that FGF23 is associated with risk for fatal and non-fatal cardiovascular events [7, 9, 11–13]. Studies looking at heart failure admissions have also shown an association with baseline FGF23 [12, 37]. We were unable to demonstrate an association between delta cFGF23 and risk for non-fatal cardiovascular events in our study. Cause of death data were not available for all patients so it was not possible to explore the association between change in cFGF23 and cardiovascular death.

The potential pathophysiological mechanisms underlying the association between cFGF23, cardiovascular events and mortality have been detailed by many recent studies [14–24], but the association between cFGF23 and non-cardiovascular death [11, 38] requires elucidation.

The study by De Bouma et al. examined the significance of change in FGF23 levels over time in relation to baseline covariates. The investigators found that an increase in time averaged FGF23 was associated with risk for cardiovascular events, progression and death but there was no association between rate of change in FGF23 and these outcomes [7]. In the study by Isakova et al. [5] trajectory of FGF23 change was significantly associated with risk for death. Our study showed a significant association between delta cFGF23 and risk for death.

It is important to recognize the limitations of our analysis. The SKS is an established cohort of referred patients with advanced CKD. The findings of our study may not apply to a non-referred CKD population or those with earlier stage disease. Our study was an observational study and as such cannot infer any causation on cFGF23 as regards the deleterious outcomes examined. We did not have vitamin D levels available for this analysis nor did we have data on heart failure events.

Despite the limitations of our analysis, we feel that our findings contribute to the literature on cFGF23 in CKD and confirm what has already been demonstrated in other cohorts. Our analysis has shown that increasing levels of cFGF23 are associated with all-cause mortality and risk for RRT but not non-fatal cardiovascular events in a largely Caucasian population with advanced CKD. There is a need for improved understanding of the factors influencing the levels of FGF23 and an increasing awareness of the pathophysiology of this biomarker to enhance our understanding of its associations with these outcomes. It is timely to consider the role that measurement of FGF23 could play in the management of CKD patients given the significant risks that are apparently associated with high and importantly increasing levels.

## Conclusion

In our study, increasing cFGF23 was significantly associated with risk for death and and RRT but not non-fatal cardiovascular events, confirming what has already been demonstrated in other cohorts.

## Supplementary Information

**Additional file 1: Supplementary Table 1.** Cox-regression models for all-cause mortality prior renal replacement therapy (univariate model)

**Additional file 2: Supplementary Table 2.** Cox-regression model for renal replacement therapy (univariate model)

**Additional file 3: Supplementary Table 3.** Cox-regression model for cardiovascular event (univariate model)

## Data Availability

The datasets used and/or analysed during the current study are available from the corresponding author on reasonable request.

## Abbreviations

FGF23: Fibroblast growth factor23
cFGF23: c-termina Fibroblast growth factor 23
CV: Cardiovascular
RRT: Renal replacement therapy
ONS: Office of National Statistics
SKS: The Salford kidney study
CRISIS: Chronic renal insufficiency standards implementation study
ELISA: Enzyme linked immunosorbent assay
MDRD: Modified Diet in Renal Disease
