# Supplementary material for: Longitudinal change in c-terminal fibroblast growth factor 23 and outcomes in patients with advanced chronic kidney disease
Source: BMC Nephrol. 2021 Oct 2;22:329. doi: 10.1186/s12882-021-02528-2 (PMC8487581; doi:10.1186/s12882-021-02528-2)
Supplement: Supplementary file 1 — Additional file 1: Supplementary Table 1. Cox-regression models for all-cause mortality prior renal replacement therapy (univariate model) [file 12882_2021_2528_MOESM1_ESM.docx]

**Supplementary Table 1 Cox-regression models for all-cause mortality prior renal replacement therapy (univariate model)**

|  | Univariate model |  |
| --- | --- | --- |
|  | HR (95% CI) | p-Value |
| Age | 1.09 (1.06-1.15) | **<0.001** |
| Male | 0.82 (0.52-1.26) | 0.367 |
| Caucasian | 4.11 (0.57-29.45) | 0.160 |
| Smoker | 1.72 (1.09-2.72) | **0.019** |
| Diabetes | 1.68 (1.07-2.64) | **0.022** |
| CVE | 2.08 (1.34-3.24) | **0.001** |
| CCF | 2.43 (1.39-4.2) | **0.002** |
| Systolic blood pressure | 1.01 (1.0-1.01) | **0.014** |
| Creatinine | 1.01 (1.0-1.01) | **<0.001** |
| MDRD eGFR | 0.94 (0.92-0.96) | **<0.001** |
| Phosphate | 5.1 (2.1- 12.6) | **<0.001** |
| Calcium | 0.18 (0.03-1.07) | 0.06 |
| Albumin | 0.92 (0.86-0.97) | **0.007** |
| Haemoglobin | 0.97 (0.95-0.98) | **<0.001** |
| PTH | 1.01 (1.01-1.01) | **<0.001** |
| CRP | 1.02 (1.01-1.04) | **0.012** |
| Urinary protein | 1.35 (0.99-1.84) | 0.058 |
| cFGF23 | 1.01 (1.01-1.02) | **<0.001** |

Model includes all baseline clinical and biochemical characteristics
