# Supplementary material for: Longitudinal change in c-terminal fibroblast growth factor 23 and outcomes in patients with advanced chronic kidney disease
Source: BMC Nephrol. 2021 Oct 2;22:329. doi: 10.1186/s12882-021-02528-2 (PMC8487581; doi:10.1186/s12882-021-02528-2)
Supplement: Supplementary file 2 — Additional file 2: Supplementary Table 2. Cox-regression model for renal replacement therapy (univariate model) [file 12882_2021_2528_MOESM2_ESM.docx]

**Supplementary Table 2 Cox-regression model for renal replacement therapy (univariate model)**

|  | Univariate model |  |
| --- | --- | --- |
|  | HR (95% CI) | p-Value |
| Age | 0.97 (0.96-0.99) | **0.001** |
| Male | 0.56 (0.36-0.88) | **0.013** |
| Caucasian | 0.69 (0.28-1.70) | 0.417 |
| Smoker | 1.05 (0.67-1.67) | 0.809 |
| Diabetes | 1.14 (0.68-1.89) | 0.622 |
| CVE | 0.65 (0.35-1.20) | 0.170 |
| CCF | 1.45 (0.72-2.92) | 0.292 |
| Systolic blood pressure | 1.00 (0.99-1.01) | 0.983 |
| Creatinine | 1.01 (1.01-1.02) | **<0.001** |
| MDRD eGFR | 0.93 (0.91-0.95) | **<0.001** |
| Phosphate | 10.8 (4.3027.2) | **<0.001** |
| Calcium | 1.51 (0.23-10) | 0.673 |
| Albumin | 0.92 (0.86-0.98) | **0.010** |
| Haemoglobin | 0.97 (0.96-0.98) | **<0.001** |
| PTH | 1.01 (1.01-1.02) | **<0.001** |
| CRP | 0.97 (0.93-1.01) | 0.173 |
| Urinary protein | 1.47 (1.09-1.98) | **0.011** |
| cFGF23 | 1.01 (1.01-1.02) | **<0.001** |

Model includes all baseline clinical and biochemical characteristics
