# Supplementary material for: Longitudinal change in c-terminal fibroblast growth factor 23 and outcomes in patients with advanced chronic kidney disease
Source: BMC Nephrol. 2021 Oct 2;22:329. doi: 10.1186/s12882-021-02528-2 (PMC8487581; doi:10.1186/s12882-021-02528-2)
Supplement: Supplementary file 3 — Additional file 3: Supplementary Table 3. Cox-regression model for cardiovascular event (univariate model) [file 12882_2021_2528_MOESM3_ESM.docx]

**Supplementary Table 3 Cox-regression model for cardiovascular event (univariate model)**

|  | Univariate model |  |
| --- | --- | --- |
|  | HR (95% CI) | p-Value |
| Age | 1.05 (1.03-1.08) | **<0.001** |
| Male | 1.24 (0.67-2.30) | 0.498 |
| Caucasian | 2.37 (0.33-17.12) | 0.39 |
| Smoker | 1.44 (0.81-2.58) | 0.21 |
| Diabetes | 1.75 (0.97-3.15) | 0.06 |
| CVE | 2.27 (1.57-4.82) | **<0.001** |
| CCF | 4.19 (2.22-7.93) | **<0.001** |
| Systolic blood pressure | 1.00 (0.98-1.01) | 0.848 |
| Creatinine | 1.01 (1.0-1.01) | **0.035** |
| MDRD eGFR | 0.97 (0.95-0.99) | **0.008** |
| Phosphate | 1.96 (0.57-6.7) | 0.281 |
| Calcium | 0.37 (0.03-4.1) | 0.425 |
| Albumin | 0.92 (0.85-1.0) | **0.05** |
| Haemoglobin | 0.97 (0.95-0.98) | **0.001** |
| PTH | 1.01 (0.99-1.01) | 0.105 |
| CRP | 1.03 (1.01-1.05) | **<0.001** |
| Urinary protein | 1.29 (0.84-1.99) | 0.234 |
| cFGF23 | 1.01 (1.0-1.02) | 0.05 |

Model includes all baseline clinical and biochemical characteristics
